# Wearable devices in palliative care for people 65 years and older: A scoping review

**DOI:** 10.1177/20552076231181212

**Published:** 2023-07-02

**Authors:** Rada Sandic Spaho, Lisbeth Uhrenfeldt, Theofanis Fotis, Ingjerd Gåre Kymre

**Affiliations:** 1 Faculty of Nursing and Health Sciences, 1786 Nord University, Bodo, Norway; 2 Danish Centre of Systematic Reviews: An Affiliate Center of The Joanna Briggs Institute, The Center of Clinical Guidelines – Clearing House, 1004 Aalborg University Denmark, Aalborg, Denmark; 3 Institute of Regional Health Research, Lillebaelt University Hospital, Southern Danish University, Kolding, Denmark; 4 School of Sport & Health Sciences, Centre for Secure, Intelligent and Usable Systems, 1947 University of Brighton, Brighton, UK

**Keywords:** Wearable electronic devices, accelerometers, actigraphy, wearable sensors, palliative care, critical illness, healthcare professionals, older people, aged, aged, 80 and over

## Abstract

**Objective:**

The objective of this scoping review is to map existing evidence on the use of wearable devices in palliative care for older people.

**Methods:**

The databases searched included MEDLINE (via Ovid), Cumulative Index to Nursing and Allied Health Literature (CINAHL) and Google Scholar, which was included to capture grey literature. Databases were searched in the English language, without date restrictions. Reviewed results included studies and reviews involving patients aged 65 years or older who were active users of non-invasive wearable devices in the context of palliative care, with no limitations on gender or medical condition. The review followed the Joanna Briggs Institute's comprehensive and systematic guidelines for conducting scoping reviews.

**Results:**

Of the 1,520 reports identified through searching the databases, reference lists, and citations, six reports met our inclusion criteria. The types of wearable devices discussed in these reports were accelerometers and actigraph units. Wearable devices were found to be useful in various health conditions, as the patient monitoring data enabled treatment adjustments. The results are mapped in tables as well as a Preferred Reporting Items for Systematic reviews and Meta-Analyses extension for Scoping Reviews (PRISMA-ScR) chart.

**Conclusions:**

The findings indicate limited and sparse evidence for the population group of patients aged 65 years and older in the palliative context. Hence, more research on this particular age group is needed. The available evidence shows the benefits of wearable device use in enabling patient-centred palliative care, treatment adjustments and symptom management, and reducing the need for patients to travel to clinics while maintaining communication with healthcare professionals.

## Introduction

It is estimated that 40 million people in the world need palliative care, but only 14% of them receive it.^1^ According to the World Health Organisation (WHO), ‘palliative care improves the quality of life of patients and that of their families who are facing challenges associated with life-threatening illness, whether physical, psychological, social or spiritual. The quality of life of caregivers improves as well.’^1^ However, several aspects of the delivery of palliative care need improvement, including access to palliative care medicines, the training of healthcare professionals, funding and cooperation between governmental and non-governmental organisations, technical assistance, the better integration of palliative care within wider healthcare systems, and enabling patients to choose whether to receive palliative care at home or in healthcare institutions.^1–3^ In 2012, the United Nations recognised that human rights related to palliative care for older people had not been sufficiently addressed. This led the Council of Europe to adopt a resolution on the ‘Promotion of human rights of older persons’ in 2014. The resolution includes a section specifically addressing palliative care, especially access to palliative care that corresponds to patients’ needs and their right to choose their own home as one of the possible care settings.^3^ Many people living with a range of chronic conditions (such as cardiovascular diseases, chronic respiratory diseases, kidney failure or diabetes) require palliative care. However, for decades, this has not been recognised in the way that, for example, the need for palliative care for cancer patients has been acknowledged.^4^ With a higher life expectancy and an increasing number of people with severe chronic diseases, more people will need palliative care in the future.^5^ In 2019, 9% of the global population was aged 65 years or above, and this percentage is predicted to increase to 16% by 2050.^6^ In Norway, in 2016, residents over the age of 65 already made up almost 17% of the population.^5^ People aged 65 and older are considered vulnerable because of their age, comorbidities, and possible care dependence.^7,8^ Older people living in rural areas may face additional difficulties in accessing healthcare services, as rural areas usually cannot offer specialised services or the same standard of healthcare services as more urban areas.^9–11^ According to the ‘Global Health Workforce Labor Market Projections for 2030’, the world will soon face a shortage of 15 million healthcare workers, which will only make the current situation worse.^12^ There is an urgent need for solutions that can meet some of these challenges.

Digital health can be one of the possible solutions to meet the aforementioned challenges. It encompasses a number of technologies, including telemedicine, telehealth, mHealth, eHealth, Internet of Things and wearable devices.^2,13,14^ Digital health increases the opportunities for personalised patient care, while at the same time reducing costs.^1,2,15^ It prevents avoidable emergency department visits by enabling timely communication with healthcare professionals.^16,17^ Healthcare professionals can monitor patients’ health and deliver care remotely, i.e. without clinical visits, whereby patients gain increased autonomy.^2,15^

Wearable devices can be defined as technological devices that are worn by the user and have sensors that can monitor various body measurements.^2,18,19^ They can be connected to mobile applications to allow a person to see the data being collected or enter reports about measurements.^2,20^ In the general adult population, there is a growing use of smartwatches, activity trackers, sleep monitors, etc.^2,20^ In healthcare, wearable devices include a range of devices that can remotely monitor a patient's blood pressure, sleep patterns, body temperature, heart rate and physical activity; they can also serve as medication reminder alarms, global positioning trackers and safety alarms and provide additional functions. Wearable devices can be used to monitor patients’ gait and track their activity, to assist in the prevention of falls for at-risk patients.^14,21,22^ They can monitor not only physiological but also sensorial and emotional states, such as depression, behavioural and/or psychological parameters and environmental parameters, such as air pollutants for people with chronic obstructive lung diseases.^18,23^ This remote monitoring allows person-centred care to be administered remotely, in patients’ homes, without requiring clinic visits.^2,14,24,25^ Healthcare professionals can react to monitored measurements from the wearable device by contacting the patient via text message about the treatment adjustments, sending reminders, making a phone call to check in with the patient in response to alarming measurements, or even sending an emergency unit to the patient's home if needed.^2,16,26^ A patient wearing a safety alarm can trigger an urgent call to healthcare professionals by pressing a button on the alarm (usually worn as an armband or a necklace). Wearable devices can monitor patients’ health status and support the provision of home care, while at the same time generating cost savings by preventing hospital visits and reducing overall hospitalisation rates.^23^

However, there are some issues with introducing wearable devices in healthcare, as there is not yet a commonly accepted classification system for wearable devices in the literature.^20,24^ Although wearable devices should be verified and validated through clinical trials, in many countries, there are no specific guidelines for the rigorous testing of wearable devices.^2,20,25,27–30^ As an example of the kind of requirements wearable devices should fulfil, we point to the evidence standards framework for digital health technologies (NICE, Tier 3b).^31^ As evidence of effectiveness, a minimum evidence standard requires high-quality intervention studies with statistically significant improvements in outcomes such as clinical measures, diagnostic accuracy, patient-reported outcomes, user satisfaction, etc.^31^ Byrom et al.^27^ suggest a framework in which wearable devices should meet criteria about safety, suitability for the intended population, validity and reliability, and additional details (acceptability, visibility, battery life, etc). According to the literature, both patients and healthcare professionals have expressed concerns related to data privacy, security, transfer and storage regarding the use of wearable devices.^15,25^ Wearable devices used in healthcare services in the European Union should comply with the General Data Protection Regulation (GDPR), although this is still not common practice.^20,32^

The use of wearable devices is increasing, but there is still a gap in the knowledge about the use of wearable devices for cancer patients in palliative care settings.^33^ Various wearable devices for monitoring physical activity are used in palliative care.^22,30,34,35^ However, evidence is still lacking regarding the wider use of wearable devices in palliative care for monitoring the aforementioned data points, such as blood pressure, blood sugar, heart rate and oxygen level. There is a particular lack of evidence within the 65 years and older population.

A preliminary search of MEDLINE, the Cochrane Database of Systematic Reviews and the *Joanna Briggs Institute Evidence Synthesis Journal* was conducted, and no published or in-progress systematic reviews or scoping reviews on the topic were identified. In MEDLINE (via Ovid), Medical Subject Headings (MeSH) terms were used: ‘palliative care AND wearable electronic devices AND (aged, OR aged, 80 and older)’. Only one result was found: A clinical trial with 10 participants. When there is insufficient evidence, a scoping review is recommended as a method, as opposed to a systematic review, which is recommended to synthesise evidence, to appraise the evidence and to inform future research and policy.^36,37^ Based on these findings, a comprehensive systematic search, following guidelines for scoping reviews, was planned and conducted to gain a full picture of the use of wearable devices in palliative care for older people. The objective of this scoping review is to map existing evidence about wearable devices that are in use in palliative care among the older population.

## Review question

*What types of wearable devices are in use for patients aged 65 years and older in palliative care?*

## Methods

This scoping review was conducted following the Joanna Briggs Institute (JBI) methodology for scoping reviews.^38,39^

## Inclusion criteria

### Participants

*This review considered studies and reviews that included patients aged 65 years and older who used wearable devices as part of their care. There were no limitations regarding gender or medical condition.*

### Concept: Wearable devices

*This review considered studies and reviews that included non-invasive wearable devices.*

### Context: Palliative care

*This review considered studies and reviews with palliative care as the context. There were no geographical limitations in the search.*

### Exclusion criteria

Studies and reviews were excluded if they: had participants with a mean/median age lower than 65 years, patients who were not receiving palliative care, wearable devices still in the testing phase (i.e. under development and not ready for the market) or the use of wearable devices with unconscious patients. Invasive wearable devices such as arterial blood pressure monitoring (which uses intravascular sensors for continuous monitoring) were excluded, as they are considered invasive.

### Types of sources of evidence

#### The search strategy

The search strategy aimed to locate both published and unpublished studies. The search strategy was not limited by the year of publication. Only sources of evidence published in the English language were included.

Box 1. Types of sources of evidence included in the search strategy.

Analytical observational studies, including prospective and retrospective cohort studies, case-control studies and analytical cross-sectional studies, and descriptive observational study designs, including case series and descriptive cross-sectional studies.^39,40^

Experimental and quasi-experimental studies that researched outcome measures, such as diagnostic accuracy, clinical, physiological measures and patient-reported outcomes, high-quality randomised controlled studies and meta-analyses of randomised controlled studies that are relevant to the social and health care system.

Qualitative studies, including but not limited to designs such as phenomenology, grounded theory, ethnography, qualitative description, action research and feminist research.^39,40^

Dissertations, reports, conference presentations, letters to the editor and systematic reviews that met the inclusion criteria.

The review followed a JBI-recommended three-step search strategy.^40^ Initially, a search for relevant literature was conducted in the Cumulative Index to Nursing and Allied Health Literature (CINAHL) database. After this initial search, words contained in titles and abstracts were analysed, and the index terms of the sources of evidence were retrieved. In the second step, the final search was done across MEDLINE via the Ovid and CINAHL databases with all identified keywords and index terms. These two databases were chosen because they enabled a search of more than 3,000 journals across a wider multidisciplinary area, rather than only the nursing field.^41–43^ The final search strategy was reviewed by a university librarian. Additionally, Google Scholar was used to identify grey literature because it can be searched for both published and unpublished (in journals) literature that might not be captured by CINAHL and MEDLINE.^44^ The search strategy, including all identified keywords and index terms, was adapted for each database and/or information source (See Table 1, Table 2, Table 3 and Table 4). The third step was searching the reference lists of the included sources of evidence. For a more systematic search, citations of all included sources of evidence were also searched following the same search strategy. The university librarian was contacted for unavailable sources of evidence, which were provided.

Box 2. Explanation of the palliative care and wearable device terms used in the search.

*The term ‘palliative care’ was used as an umbrella covering all care for life-limiting illnesses. Palliative care encompasses terminal and end-of-life care as consecutive parts since the timing of death is unpredictable.^5,45–50^ Terminal care as a MeSH term encompasses the medical and nursing care of patients in the terminal stage of an illness.^51^ End-of-life care focuses on care for people who are in the last days or months of their life.^45,52^

*‘Wearable devices’ is a MeSH term (‘wearable electronic devices’ for MEDLINE and ‘wearable sensors’ for the CINAHL database).^19^ This term describes our specific point of interest and is already used in health-related articles.^20,25,32,33,35^

**Table 1.** Cumulative Index to Nursing and Allied Health Literature (CINAHL) database with Medical Subject Headings MeSH terms and keywords search for ‘wearable devices in palliative care for older people’.

Wearable Devices	Palliative Care	Older People 65+
Mesh Terms	Mesh Terms	Mesh Terms
Wearable sensors (CH) Equipment alarm systems(CH) Security measures, Electronic(CH) Wireless communications (CH) Signal processing, computer-assisted(CH) Monitoring, physiologic (CH) Home diagnostic tests (CH) Monitoring, direct pressure(CH) Blood glucose self-monitoring(CH) Blood pressure monitoring, ambulatory (CH) Product surveillance (CH) for GPS tracking Digital technology (CH) Polysomnography (CH) Actigraphy (CH) Emergency medical tags (CH)	Palliative care Terminally ill patients Critically ill patients Critical illness Terminally ill Life support care Cancer patients Oncologic care Oncology Terminal Care (SABA) Terminal care Hospice care Hospice patients	Aged (CH) Aged 80 and over Frail elderly
Keywords	Keywords	Keywords
Sensor Wearable Smart wristbands Armband Bracelet Photoplethysmography Sleep tracker Smartwatch Fitness trackers Smart clothing Pedometer Galvanic Skin Response (GSR) Pain relief Wearable Pulse oximeter Sleep monitor smart ring EARRING backing Monitoring Alarm	Dying patient End-of-life care (Palliative OR terminal OR ‘end of life’) ADJ3 (care OR treatment) Dying (palliative OR terminal OR ‘end of life’) W2 (care OR treatment)	Aged Aged 80 and over Elderly Elderly.tw. Older people Older adult Senior Geriatric Older W2 people Older N2 people Older patient Older person
Additions		
Wearable N2 (device OR sensor OR technology) Wearable ADJ3 (device OR sensor OR technology) GPS tracking GPS technology GPS tracking motion sensors GPS W3 sensors		

The search was conducted 19 November 2021, producing 623 results.

**Table 2.** Full search strategy for the Cumulative Index to Nursing and Allied Health Literature database.

#	Searches	Results
1	Aged OR Aged 80 and over OR Frail elderly OR Elderly OR Elderly.tw. OR Older people OR Older adult OR Senior OR Geriatric OR Older W2 people OR Older N2 people OR Older patient OR Older person OR	1,042,200
2	Wearable sensors OR Equipment Alarm Systems OR Security Measures, Electronic OR Wireless communications OR Signal Processing, Computer-Assisted OR Monitoring, Physiologic OR Home Diagnostic Tests OR Monitoring, Direct Pressure OR Blood Glucose Self-Monitoring OR *Blood Pressure Monitoring, Ambulatory* OR Product Surveillance OR Global Positioning System OR Digital technology OR Polysomnography OR Actigraphy OR Pedometers OR *Emergency Medical Tags* OR Sensor OR Wearable OR Smart wristbands OR Armband OR Bracelet OR Photoplethysmography OR Sleep tracker OR Smartwatch OR Fitness trackers OR Smart clothing OR Pedometer OR Galvanic Skin Response OR Pain relief OR Wearable pulse oximeter OR Sleep monitor OR Smart ring OR Earring backing OR Monitoring OR Alarm OR Wearable N2 (device OR sensor OR technology) OR GPS tracking OR GPS technology OR GPS tracking motion sensor* OR GPS W3 sensor* OR	139,463
3	Palliative care Terminally Ill Patients Critically Ill Patients Critical Illness Terminally ill Life support care Cancer patients Oncologic care Oncology Terminal care (SABA) Terminal care Hospice care Hospice patients Dying patient End-of-life care (Palliative OR terminal OR ‘end of life’) ADJ3 (care OR treatment) Dying (palliative OR terminal OR ‘end of life’) W2 (care OR treatment)	161,446
4	1 AND 2 AND 3	623

The search was conducted on 19 November 2021.

**Table 3.** Full search strategy for MEDLINE via the Ovid database.

#	Searches	Results
1	Wearable electronic devices	4,983
2	Monitoring, ambulatory	8,525
3	clinical alarms/ or ‘electrical equipment and supplies’/or emergency medical tags/ or protective devices/ or self-help devices/	15,018
4	Wireless technology	4,062
5	Blood pressure monitoring, ambulatory	10,767
6	Blood glucose self-monitoring	7,984
7	Remote sensing technology	3,461
8	Digital technology	359
9	Polysomnography	23,192
10	Actigraphy	4309
11	sensor*.mp. [mp = title, abstract, original title, name of substance word, subject heading word, floating sub-heading word, keyword heading word, organism supplementary concept word, protocol supplementary concept word, rare disease supplementary concept word, unique identifier, synonyms]	354,947
12	track*.mp. [mp = title, abstract, original title, name of substance word, subject heading word, floating sub-heading word, keyword heading word, organism supplementary concept word, protocol supplementary concept word, rare disease supplementary concept word, unique identifier, synonyms]	139,960
13	wearable.mp. [mp = title, abstract, original title, name of substance word, subject heading word, floating sub-heading word, keyword heading word, organism supplementary concept word, protocol supplementary concept word, rare disease supplementary concept word, unique identifier, synonyms]	11,852
14	smart wristband*.mp. [mp = title, abstract, original title, name of substance word, subject heading word, floating sub-heading word, keyword heading word, organism supplementary concept word, protocol supplementary concept word, rare disease supplementary concept word, unique identifier, synonyms]	13
15	armband*.mp. [mp = title, abstract, original title, name of substance word, subject heading word, floating sub-heading word, keyword heading word, organism supplementary concept word, protocol supplementary concept word, rare disease supplementary concept word, unique identifier, synonyms]	554
16	bracelet?.mp. [mp = title, abstract, original title, name of substance word, subject heading word, floating sub-heading word, keyword heading word, organism supplementary concept word, protocol supplementary concept word, rare disease supplementary concept word, unique identifier, synonyms]	477
17	photopletismography.mp. [mp = title, abstract, original title, name of substance word, subject heading word, floating sub-heading word, keyword heading word, organism supplementary concept word, protocol supplementary concept word, rare disease supplementary concept word, unique identifier, synonyms]	13
18	sleep tracker?.mp. [mp = title, abstract, original title, name of substance word, subject heading word, floating sub-heading word, keyword heading word, organism supplementary concept word, protocol supplementary concept word, rare disease supplementary concept word, unique identifier, synonyms]	43
19	smartwatch*.mp. [mp = title, abstract, original title, name of substance word, subject heading word, floating sub-heading word, keyword heading word, organism supplementary concept word, protocol supplementary concept word, rare disease supplementary concept word, unique identifier, synonyms]	370
20	smart watch*.mp. [mp = title, abstract, original title, name of substance word, subject heading word, floating sub-heading word, keyword heading word, organism supplementary concept word, protocol supplementary concept word, rare disease supplementary concept word, unique identifier, synonyms]	100
21	fitness tracker*.mp. [mp = title, abstract, original title, name of substance word, subject heading word, floating sub-heading word, keyword heading word, organism supplementary concept word, protocol supplementary concept word, rare disease supplementary concept word, unique identifier, synonyms]	1,010
22	smart cloth*.mp. [mp = title, abstract, original title, name of substance word, subject heading word, floating sub-heading word, keyword heading word, organism supplementary concept word, protocol supplementary concept word, rare disease supplementary concept word, unique identifier, synonyms]	58
23	pedometer*.mp. [mp = title, abstract, original title, name of substance word, subject heading word, floating sub-heading word, keyword heading word, organism supplementary concept word, protocol supplementary concept word, rare disease supplementary concept word, unique identifier, synonyms]	2,511
24	galvanic skin response.mp. [mp = title, abstract, original title, name of substance word, subject heading word, floating sub-heading word, keyword heading word, organism supplementary concept word, protocol supplementary concept word, rare disease supplementary concept word, unique identifier, synonyms]	8,682
25	pain relief.mp. [mp = title, abstract, original title, name of substance word, subject heading word, floating sub-heading word, keyword heading word, organism supplementary concept word, protocol supplementary concept word, rare disease supplementary concept word, unique identifier, synonyms]	29,229
26	wearable pulse oximeter.mp. [mp = title, abstract, original title, name of substance word, subject heading word, floating sub-heading word, keyword heading word, organism supplementary concept word, protocol supplementary concept word, rare disease supplementary concept word, unique identifier, synonyms]	12
27	sleep monitor*.mp. [mp = title, abstract, original title, name of substance word, subject heading word, floating sub-heading word, keyword heading word, organism supplementary concept word, protocol supplementary concept word, rare disease supplementary concept word, unique identifier, synonyms]	696
28	smart ring.mp. [mp = title, abstract, original title, name of substance word, subject heading word, floating sub-heading word, keyword heading word, organism supplementary concept word, protocol supplementary concept word, rare disease supplementary concept word, unique identifier, synonyms]	6
29	earring backing.mp. [mp = title, abstract, original title, name of substance word, subject heading word, floating sub-heading word, keyword heading word, organism supplementary concept word, protocol supplementary concept word, rare disease supplementary concept word, unique identifier, synonyms]	2
30	alarm.mp. [mp = title, abstract, original title, name of substance word, subject heading word, floating sub-heading word, keyword heading word, organism supplementary concept word, protocol supplementary concept word, rare disease supplementary concept word, unique identifier, synonyms]	9,537
31	1 or 3 or 4 or 5 or 6 or 7 or 8 or 9 or 10 or 11 or 12 or 13 or 14 or 15 or 16 or 17 or 18 or 19 or 20 or 21 or 22 or 23 or 24 or 25 or 26 or 27 or 28 or 29 or 30	606,416
32	Palliative Care	58,645
33	Terminal Care/ or Terminally Ill	35,160
34	Critical Illness	34,096
35	Critical Care	56,663
36	Life Support Care	7844
37	Medical Oncology	21,012
38	Terminal Care	30,168
39	Hospice Care	7,193
40	dying patient*.mp. [mp = title, abstract, original title, name of substance word, subject heading word, floating sub-heading word, keyword heading word, organism supplementary concept word, protocol supplementary concept word, rare disease supplementary concept word, unique identifier, synonyms]	2,948
41	end of life care.mp. [mp = title, abstract, original title, name of substance word, subject heading word, floating sub-heading word, keyword heading word, organism supplementary concept word, protocol supplementary concept word, rare disease supplementary concept word, unique identifier, synonyms]	10,502
42	32 or 33 or 34 or 35 or 36 or 37 or 38 or 39 or 40 or 41	197,698
43	‘Aged, 80 and over’/ or Aged/	3,332,583
44	Frail Elderly	13,288
45	geriatric.mp. [mp = title, abstract, original title, name of substance word, subject heading word, floating sub-heading word, keyword heading word, organism supplementary concept word, protocol supplementary concept word, rare disease supplementary concept word, unique identifier, synonyms]	76,620
46	elderly.mp. [mp = title, abstract, original title, name of substance word, subject heading word, floating sub-heading word, keyword heading word, organism supplementary concept word, protocol supplementary concept word, rare disease supplementary concept word, unique identifier, synonyms]	246,016
47	older W1 people.mp. [mp = title, abstract, original title, name of substance word, subject heading word, floating sub-heading word, keyword heading word, organism supplementary concept word, protocol supplementary concept word, rare disease supplementary concept word, unique identifier, synonyms]	0
48	older people.mp. [mp = title, abstract, original title, name of substance word, subject heading word, floating sub-heading word, keyword heading word, organism supplementary concept word, protocol supplementary concept word, rare disease supplementary concept word, unique identifier, synonyms]	28,975
49	older adult.mp. [mp = title, abstract, original title, name of substance word, subject heading word, floating sub-heading word, keyword heading word, organism supplementary concept word, protocol supplementary concept word, rare disease supplementary concept word, unique identifier, synonyms]	7,848
50	senior.mp. [mp = title, abstract, original title, name of substance word, subject heading word, floating sub-heading word, keyword heading word, organism supplementary concept word, protocol supplementary concept word, rare disease supplementary concept word, unique identifier, synonyms]	31,626
51	older person*.mp. [mp = title, abstract, original title, name of substance word, subject heading word, floating sub-heading word, keyword heading word, organism supplementary concept word, protocol supplementary concept word, rare disease supplementary concept word, unique identifier, synonyms]	11,429
52	43 or 44 or 45 or 46 or 47 or 48 or 49 or 50 or 51	3,408,522
53	31 and 42 and 52	1220

The search was conducted on 20 November 2021.

**Table 4.** Full search strategy for Google Scholar; search retrieved on 1 December 2021.

Keywords	Wearable, palliative, older, sensor
Limitations	Any date, sort by relevance, Any language, Any type: review articles, include patents, include citations
Where my words occur	Anywhere in the article
First 20 pages	200 results

#### Study selection

Following the search, all identified sources of evidence were uploaded into the RAYYAN web tool^53^ and duplicates were removed. Two of the authors (RSS and IGK) screened the titles, abstracts and full articles independently using pre-defined inclusion and exclusion criteria. The reasons for the exclusion of sources of evidence that did not meet the inclusion criteria were recorded and reported in the scoping review. Sources in which it was unclear whether or not patients were receiving palliative care were excluded, or the authors were contacted for clarification.^54,55^ Disagreements that arose between the reviewers at each stage of the selection process were resolved through discussion and with additional reviewers (TF and LU). As an example, sources of evidence concerning patients on mechanical ventilation in intensive care units were excluded, even though wearable devices were used, as they were outside our inclusion criteria and against our aim (the use of wearable devices with unconscious patients). As mentioned previously, the two examples of guidelines for wearable devices, NICE^31^ and Byrom et al.,^27^ have stricter requirements than this review. For the purposes of this review, the only exclusions relating to the devices themselves were where devices were in the testing phase (under development and not ready for the market). In addition, the Preferred Reporting Items for Systematic reviews and Meta-Analyses extension for Scoping Reviews (PRISMA-ScR) flow diagram facilitated the reporting of this process.^56^

#### Data extraction

Data extracted from the sources of evidence included in the scoping were reviewed independently by three of the authors (RSS, IGK and LU) using a data extraction tool developed by the reviewers. The fourth author (TF) was consulted in all phases of the scoping review, from the selection of keywords, to decisions on which types of wearable devices to include, to data extraction. The authors of the sources were contacted to request missing or additional data, where required.^57–62^ The extracted data of the included sources of evidence (further: reports) are presented in the table with authors, year of publication, country where the research was conducted, participants’ characteristics, wearable devices, palliative care contexts and other details in Table 5 and Table 6.

**Table 5.** Mapping overview of the studies identified (number = 5).

Author and country	Participants characteristics	Wearable device/ *manufacturer`s name*	Palliative care context and Location	Study aim	Study type and study duration	Study results	Study conclusion	What kind of wearable devices are in use in palliative care for older people 65 years and older?
Froggatt et al.; UK^63^	Patients with advanced dementia (n = 32) Mean age 81.5 years males (n = 17) females (n = 15)	Actigraph/ *ActiGraph*^a^	Nursing homes in England providing care for people with dementia in end-of-life care	To ascertain the feasibility of conducting a full trial of the *Namaste Care intervention*	Feasibility study 24 weeks	The data demonstrated that this population is inactive, the sleep patterns are not healthy, and sleep is fragmented	Collecting data with ActiGraph is feasible ActiGraph can measure variations within a day and between days for the same participant The actiGraph device should be used on the wrist and not on the ankle because of better visibility of possible bruising	Actigraph was used for monitoring physical activity and sleep pattern
Godfrey et al.; Ireland^64^	Patients in delirium receiving palliative care (n = 40) Mean age 68.4 years males (n = 23) females (n = 17)	Accelerometer/ *ActivPAL*^b^ and analogue accelerometer-based device^c^	Hospice palliative care centre	To determine motoric subtypes in patients with delirium To compare two accelerometers’ measurements and to estimate the feasibility of accelerometry- based monitoring	Feasibility study 24 h	The data from accelerometers provided data for software calculations Software data correctly classified delirium-specific checklists for 87% of the patients tested	Both accelerometer devices provided validated data Accelerometry measurements are a reliable and feasible method of continuous monitoring Accelerometry data can be used to objectively measure body activity	An accelerometer was used to monitor physical activity and classify patients according to activity level
Godfrey et al.; Ireland^65^	Patients in palliative care, divided into two groups: patients with delirium and a palliative control group of patients (total number of all patients n = 34) Patients with delirium group(n = 25) Mean age not available Most patients were above 65 years males (n = 16), females (n = 9) Palliative control group patients (n = 9) mean age 65.3 years males (n = 6), females (n = 3)	Accelerometer/ *ActivPAL* *Professional*^b^	Hospice Palliative care centre	To develop software analysis (classification tree) for subtyping patients with delirium into hyperactive, hypoactive and mixed groups	Cross-sectional study 24 h	A protocol for classifying delirium subtypes was developed A high level of classification accuracy (92.3%) was achieved Accelerometer measurements were useful for monitoring the mobility of delirium patients and determining delirium subtypes as hyperactive, mixed and hypoactive The mobility levels weren`t different for hyperactive/mixed subtypes between delirium and control palliative group patients	Accelerometer-based monitoring can be used together with classification tree system training to estimate the subtype mobility levels of delirium patients	An accelerometer was used to measure physical activity
Maddocks and Wilcock; UK^66^	Patients with thoracic cancer (n = 84) Mean age 66 years males (n = 54) females (n = 30)	Accelerometer/ *ActivPAL*^b^	Oncology outpatient clinics from the UK	To monitor patients’ physical activity and compare it with estimated ECOG performance status	Collated data from 3 studies that used ActivPal 1 week	Physical activity measurements differed significantly in comparison with and between the performance status categories of patients	Objective physical activity measuring is more sensitive than the estimation of ECOG performance status; This is important for patients with cachexia for better patient-centred care	An accelerometer was used to measure physical activity
Suibkitwanchai et al.; UK^67^	Patients with advanced dementia (n = 26) demographic data are not available and 14 non-palliative participants without demographic data	Wrist-worn accelerometer/ *GENEActiv*^d^	Nursing homes in England providing care for people with dementia in end-of-life care	To study, test and provide a detailed methodology for four different statistical analyses To compare them, and show the relationships between them through analysis from statistical measurements taken from people with and without dementia	Cross-sectional study Maximum 28 days	The data from patients with dementia showed that there is no clear circadian cycle in this group, as it was in the control group; using 5-minute subsampling intervals showed more intradaily variability than historically used hourly subsampling	The four statistical analyses found that patients with advanced dementia do not have significant behavioural changes between day and night time; intradaily variability should be calculated every 5 min, but not more frequently, as it would worsen performance. There is potential to gain insight into sleep and activity patterns, and their disruption with the use of high-frequency accelerometry measurements	An accelerometer was used to measure physical activity and sleep patterns

ECOG: Eastern Cooperative Oncology Group.

^a^ ActiGraph (Activinsights Ltd, Kimbolton, UK).

^b^ ActivPAL (PAL™ Technologies Ltd, Glasgow, UK).

^c^ Analog accelerometer-based device (Analog Devices BV, Limerick, Ireland).

^d^ GENEActiv (Activinsights Ltd, Kimbolton, UK).

**Table 6.** Mapping overview of the studies/review identified (number = 1).

Author and country	Participants	Wearable device/ manufacturer`s name	Care context	Review aim	Review method	Databases searched	Review conclusion	Number of studies matching inclusion criteria	Matching study author, year, country	Review the conclusion for the matching study	Summarised details for the review
Chan et al. Canada^65^	People 65 years and older (n = 10–1324) Mean age 66 to 84.2 years	Accelerometer/ActivPAL	-One residential aged care, -six hospital in-patient clinics, -nine outpatient clinics (2 from cancer clinics) -eight community-dwellers -two palliative clinics (outpatient and hospice)	To scope and do a quality assessment of published literature on physical activity patterns of older people who used the activPAL activity monitor. Quality assessment was done using Quality Assessment and Validity Tool (QAVT)	Rapid review	17 databases CINAHL, MEDLINE, and SPORTDiscus were screened and activPAL bibliography The report included 24 studies published between 2007–2015	There is a lack of high-quality studies on using ActivPal by older people in long-term care. ActivPal is a feasible device for activity monitoring.	2	Maddocks and Wilcock^66^ UK Godfrey et al.; Ireland^64^	Quality assessment for the two included studies was that they were low quality. From 24 studies, included in the report, 7 were assessed as low quality and the remaining 17 were assessed as medium quality	Of 24 included reports, only 2 matched our inclusion criteria, and are presented in Table 5. Both used accelerometers as a wearable devices.

CINAHL: Cumulative Index to Nursing and Allied Health Literature

## Results

**Figure 1.**
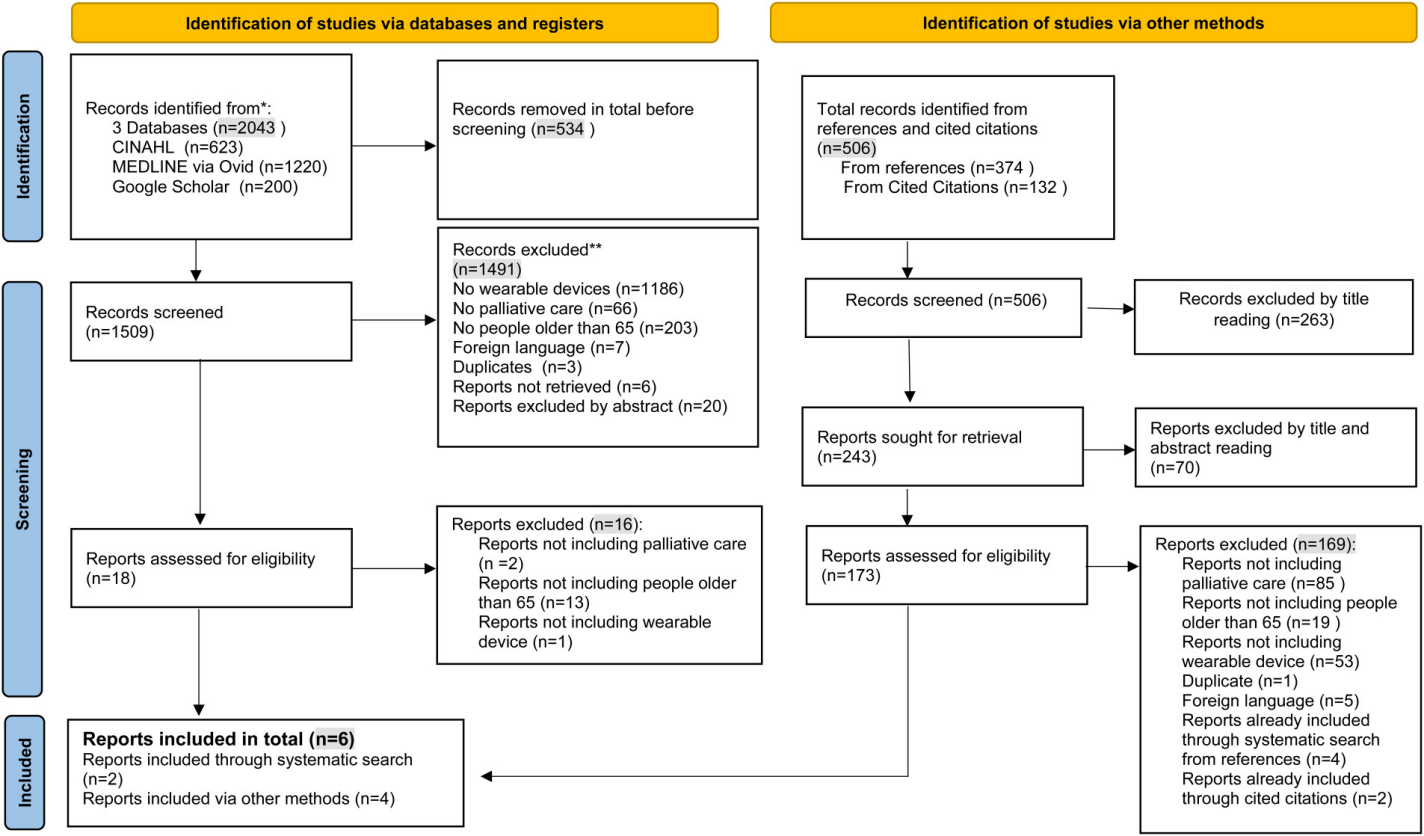
Preferred Reporting Items for Systematic reviews and Meta-Analyses extension for Scoping Reviews 2020 flow diagram.^56^

The full evidence mapping process is presented in the following PRISMA-ScR flow diagram^56^ in Figure 1. The initial search yielded 2,043 results. After duplicates were removed, the remaining 1,509 results were screened by abstract, title and keywords. The most common reasons for exclusion at this stage were the absence of the concept of wearable devices (n = 1186), participant populations that did not meet the mean age minimum of 65 (n = 203), and research contexts outside of a palliative context (n = 66). With these sources of evidence excluded, 38 remained, 18 of which were assessed for eligibility. Of these 18 reports, 16 were excluded because the mean/median participant age was under 65 (n = 13), there was no reference to palliative care (n = 2) or no wearable devices were used (n = 1).

The remaining two reports included one review and one feasibility study. Our reference search of these two reports yielded three more sources, which met our inclusion criteria and were added to the final pool. For a more comprehensive search, we also searched for other sources, which had cited any of these five reports, and retrieved one more report, for a final total of six. The most interesting and relevant findings of the included reports connected to the research question, considered the types of wearable devices and the patients involved in the studies.

## The included reports

The types of wearable devices discussed in the included reports were accelerometers and actigraph units. ActivPAL™ accelerometers were used in three studies^63,66,68^ and wrist-worn GENEActiv accelerometers were used in one.^67^ ActiGraph products (produced by Activinsights Ltd, Kimbolton, UK) were used in one study.^64^ Both types of wearable devices were used to objectively measure physical activity and sleep pattern monitoring. Three studies tested software for interpretation of the data from wearable devices; two of these led to the development of the correct assessment of physical activity,^63,68^ while the third led to a proposal for a different frequency of reading the monitored data (subsampling). This one differed by dropping the subsampling from the previously used 1 hour to a 5-minute subsampling of the measurements.^67^ The data from wearable devices provided more accurate variations in physical activity and therefore allowed for individualised treatments. Physical activity was connected to better symptom management and life quality for palliative oncology patients.^66^

The patients that participated in the included studies were patients with dementia in end-of-life care,^64,67^ cancer patients^66^ and delirium patients.^63,68^ Studies that measured physical activity helped develop new treatments and led to adjustments in treatment for patients with different activity levels in delirium.^63,68^ The accelerometric data collected on delirium patients in the included studies discovered three groups of delirium patients: hyperactive, hypoactive and mixed groups. With new insight into levels of physical activity, treatment could be adjusted based on the above listed groups.^63,68^ Longer studies (i.e. those lasting 20 days or more) showed that wearable devices had good patient acceptability.

The sample size in the included reports ranged from 32 to 84 participants, with a total of 216 participants aged 65 or over, with a mean age range of 65.3–81.5 years. Demographic data beyond age were available for four studies,^63,64,66,68^ which included, in total, 116 male and 74 female participants. Of the six included reports, three were studies from the UK,^64,66,67^ two were studies from Ireland^63,68^ and one was a rapid review from Canada.^65^ These sources were published between 2009 and 2020. Detailed results are presented in Table 5 and Table 6.

All the studies showed that using wearable devices is feasible and beneficial for patient outcomes. They also provided patient-centred, individualised care, with treatments adjusted for each patient according to the measurements.

## Discussion

Considering the number of people in need of palliative care, the obstacles in delivering palliative care and the possibilities that wearable devices can offer, we searched for existing sources of evidence on the use of wearable devices in palliative care for older people. The literature shows that wearable devices can improve patient monitoring and provide data to facilitate treatment adjustments through communication with healthcare professionals without unnecessary clinical visits, thereby supporting the provision of individualised, patient-centred care.^18,20,26,69^ In this scoping review, we found only six sources of evidence, using two types of wearable devices: accelerometers and actigraph units.

In the study by Jensen et al.,^70^ palliative cancer patients wore ‘SenseWear®’ wristband devices, which were used to continuously and objectively monitor physical activity, sleep patterns and energy expenditure to study the effects of physical activity on cancer-related symptoms through physical activity programs. The study concluded that a physical activity program was feasible since the fatigue and pain levels decreased in cancer patients in palliative care, and their sleep duration increased. This finding correlates with that of the study by Maddocks and Wilcock.^66^ This highlights the potential for accelerometers to provide objective data, but also the need to adjust the level of physical activity according to each patient's performance status, as well as other factors. In this way, wearable devices support treatment adjustments according to the individual patient's needs.

Similarly, the study on the use of ActivPAL accelerometers with palliative oncology patients by Ferriolli et al.^71^ showed a correlation between physical activity, quality of life and disease stage. In contrast, the study of Skipworth et al.'s^72^ of physical activity in advanced cancer patients using an ActivPal accelerometer did not find it useful for patients whose cancer was so advanced that they were unable to independently care for themselves (with low-performance status). However, Froggatt et al. studied end-of-life dementia patients using actigraph units and found that the patients were not an active population and had disturbed sleep patterns. These data, together with the introduction of other interventions as part of the ‘Namaste’ program, not only helped to improve patient treatment but also led to improved satisfaction among healthcare professionals and family members.^64^ This shows that following the patient's needs, and adjusting to them, makes it possible to satisfy not only the patient but also their family and healthcare professionals.

Not all advanced-stage palliative patients require the same treatment or the same amount of physical activity. Actigraph units were used with cancer patients in a study by Fujisawa et al. and showed potential in predicting survival and quality of life.^73^ They used a wrist-worn actigraph variant, Actiwatch, to measure physical activity and sleep and rest periods in lung cancer patients. Their activity level and quality of life correlated with the stage of the disease and performance status, similar to the findings of Maddocks and Willcock's study.^66,73^ With objective measurements using wearable devices, it is possible to adjust treatments according to individual needs. Remote monitoring, which wearable devices can provide, enables treatment adjustments without clinical visits through active communication with healthcare professionals.

Activity trackers seem to be the most frequently used type of wearable device in research involving older people, not only in the studies included here but also in others, like Beekman et al. and Brickwood et al.^74,75^ The use of commercial wearable devices, like Fitbit, was also studied together with patient-reported outcomes about their symptoms through a mobile application. The study, which involved 10 palliative gynaecology patients with a mean age of 60 years, showed improved communication between patients and healthcare professionals, which led to better symptom management.^76^ Accelerometers and actigraph units can provide information about not only physical activity but also sleep patterns. Patients with advanced dementia and delirium can experience disturbed sleep patterns,^64,68^ and data from activity-monitoring devices can help determine patient-centred treatment adjustments.

As mentioned above, wearable devices used in healthcare can also measure heart and respiration rates, skin temperature, body posture, and activity as well as detect falls.^77^ Lahens et al. suggest that some devices can measure all this, as well as caloric burn, blood oxygen level (SpO2), fatigue and other data points, which are useful measurements for patients with chronic renal insufficiency.^78^ Gait monitoring and fall prevention are also possible with wearable devices, as shown in a study by Zhou et al.^79^ There are indications that the use of wearable devices that track gait can be used for the early diagnosis of cognitive frailty, together with a quick test of walking and counting backwards. Such a test lasts about a minute and produces a significant amount of useful information and possible diagnoses. Patients with cognitive frailty have deteriorated gait, and their gait metrics are altered (not just gait speed but also gait cycle, gait unsteadiness, etc.). These patients may have a heightened concern about falling and consequently less physical activity.^79^ With the use of wearable devices that can warn patients about gait change and predict a fall, these patients can stay physically active for longer, have fewer falls and experience a better quality of life.

Patients in palliative care are considered a vulnerable group, and as mentioned above, palliative care includes people with a variety of chronic conditions.^4,80^ Four of the examined studies included patients whose capacity to give informed consent was potentially compromised due to severe dementia or delirium.^63,64,67,68^ These groups of patients are hard to recruit for research. Therefore, the findings from these studies are even more valuable. However, a question arises about other palliative patient groups aged 65 and older, such as diabetic, cardiovascular and other palliative patients with comorbidities. Since there is evidence of the use of wearable devices for monitoring heart rate, blood sugar, oxygen level, etc.,^81^ why are older palliative patients left out? There is evidence that wearable devices can prevent avoidable emergency visits and deliver remote care.^2,15–17^ The future will likely bring shortages in healthcare workers,^12^ and the WHO is promoting the digitalisation of health systems.^13^ Against this backdrop, there is a need for more evidence of research on this group, i.e. patients (65 and older) in palliative care. The evidence from the literature illustrates the potential benefits of the wider use of wearable devices in practice, rather than solely in a research context. One of the examples is GPS trackers, which are used as wearable devices in everyday life to locate people with dementia, allowing them to be found if they get lost.^82,83^

Nevertheless, the issues surrounding the introduction of wearable devices in health systems remain. As mentioned above, standardisation, guidelines, and other uniform regulations are still missing,^2,20,27^ which can partially explain the gap in literature. On the other hand, these issues highlight the future work needed to enable the wider use of wearable devices.

The results of this scoping review show that the evidence is limited in this specific palliative context and population. However, the evidence that exists demonstrates that wearable devices can be effective in monitoring and communicating patients’ needs, and should be in wider use in the future.

## Strengths and limitations

The MeSH term ‘wearable electronic devices’ was introduced in 2018.^19^ It seems that the use of the term in our search pool was late to emerge compared to its use in the industry, since none of the sources of evidence in our scoping review used MeSH terms containing the words ‘wearable’ or ‘sensor’. On the contrary, other keywords, rather than the MeSH terms, led us to the reports presented in this scoping review. The diversity of terms used by researchers reflects the novelty of the area we are exploring and the plurality of the technologies involved. This scoping review has strength in that it was a comprehensive systematic search, which included as many keywords as possible, alongside the MeSH terms, to cover the body of research on the topic. Although the goal of a scoping review is normally only mapping the existing evidence, we provided more examples of the use of wearable devices to show the possibilities that lie ahead.

The limitations of this scoping review include the lack of detailed information in the searched literature concerning our research question, particularly in terms of lacking details about participants’ characteristics. There is also a possibility that some sources of evidence might not have been covered in this search because many sources used different names for the devices used in the research rather than the MeSH terms related to wearable devices. The use of many keywords contributed to overcoming this limitation. Another limitation may be the exclusion of studies of wearable devices in the testing/developmental phase, as this can be considered to have eliminated a large body of literature. To mitigate this, we checked whether those wearable devices reached and became available in the market, and if no such data were found, the relevant artiles were excluded since these devices are not available anymore. In some cases, the palliative context was not reported, or it was not clear whether or not the context was palliative at all. Where possible, authors were contacted for clarification about details, but the response rate was very low. Cooperation between the wearable device manufacturers and clinical researchers could be beneficial for future studies since most of the engineering reports in our search had details about the wearable devices but insufficient participant details.

We did not assess the quality of the included studies, but it is noteworthy that accelerometers and actigraph units were used only for monitoring physical activity and sleep, even though these devices can offer much more data, as presented above. Despite this limitation, the field of wearable devices is developing quickly, so we can expect more data to be available in the future.

## Conclusions

The evidence on the use of wearable devices in palliative care for patients older than 65 is limited. As a result, there is a need for more research focusing on this age group in a palliative context. The existing evidence on the use of wearable devices in the palliative context shows the benefits of their use by enabling patient-centred care, better physical activity monitoring, improved quality of life, fall prevention, treatment adjustments, less unnecessary travelling for clinic visits, etc. Our search found studies of two types of wearable devices: accelerometers and actigraph units.

## Future recommendations

Wearable devices are an emerging technology that promises many useful opportunities in healthcare. Their continued development will likely increase their applicability in healthcare, giving patients options for better patient-centred care in places of their choice, while encouraging healthier habits. Wearable devices may provide better patient monitoring and facilitate treatment adjustments by healthcare professionals, while at the same time improving cost-effectiveness and requiring fewer healthcare professionals involved in care. As time progresses, we expect that new developments in wearable technology will enable more and more possibilities for patients and healthcare professionals.

We argue that research is important and necessary for the population of people older than 65, especially in palliative care, where wearable devices can be beneficial. Still, their potential has yet to be fully explored. Future avenues would suggest more research on different types of wearable devices and their benefits, particularly for older populations in palliative ­care. Future studies should also focus on providing more detailed reporting.

## Supplemental Material

sj-docx-1-dhj-10.1177_20552076231181212 - Supplemental material for Wearable devices in palliative care for people 65 years and older: A scoping review Click here for additional data file. Supplemental material, sj-docx-1-dhj-10.1177_20552076231181212 for Wearable devices in palliative care for people 65 years and older: A scoping review by Rada Sandic Spaho, Lisbeth Uhrenfeldt, Theofanis Fotis and Ingjerd Gåre Kymre in DIGITAL HEALTH

## List of abbreviations

WHO: World Health Organisation
NICE: Evidence standards framework for digital health technologies
GDPR: General Data Protection Regulation
UN: United Nations
CINAHL: Cumulative Index to Nursing and Allied Health Literature
ECOG: Eastern Cooperative Oncology Group
PS: Performance status
JBI: Joanna Briggs Institute
PRISMA-ScR: Preferred Reporting Items for Systematic reviews and Meta-Analyses extension for Scoping Reviews.
